# Configurational Molecular Glue: One Optically Active Polymer Attracts Two Oppositely Configured Optically Active Polymers

**DOI:** 10.1038/srep45170

**Published:** 2017-03-24

**Authors:** Hideto Tsuji, Soma Noda, Takayuki Kimura, Tadashi Sobue, Yuki Arakawa

**Affiliations:** 1 Department of Environmental and Life Sciences, Graduate School of Engineering, Toyohashi University of Technology, Tempaku-cho, Toyohashi, Aichi 441-8580, Japan

## Abstract

D-configured poly(D-lactic acid) (D-PLA) and poly(D-2-hydroxy-3-methylbutanoic acid) (D-P2H3MB) crystallized separately into their homo-crystallites when crystallized by precipitation or solvent evaporation, whereas incorporation of L-configured poly(L-2-hydroxybutanoic acid) (L-P2HB) in D-configured D-PLA and D-P2H3MB induced co-crystallization or ternary stereocomplex formation between D-configured D-PLA and D-P2H3MB and L-configured L-P2HB. However, incorporation of D-configured poly(D-2-hydroxybutanoic acid) (D-P2HB) in D-configured D-PLA and D-P2H3MB did not cause co-crystallization between D-configured D-PLA and D-P2H3MB and D-configured D-P2HB but separate crystallization of each polymer occurred. These findings strongly suggest that an optically active polymer (L-configured or D-configured polymer) like unsubstituted or substituted optically active poly(lactic acid)s can act as “a configurational or helical molecular glue” for two oppositely configured optically active polymers (two D-configured polymers or two L-configured polymers) to allow their co-crystallization. The increased degree of freedom in polymer combination is expected to assist to pave the way for designing polymeric composites having a wide variety of physical properties, biodegradation rate and behavior in the case of biodegradable polymers.

Poly(l-lactic acid) (L-PLA) (Fig. 1) is a optically active bio-based and biodegradable polyester which can be produced from renewable resources such as starch^1^,^2^,^3^,^4^,^5^,^6^,^7^,^8^,^9^,^10^. PLLA and their copolymers are utilized for biomedical, pharmaceutical, and environmental applications, because of their biodegradability and very low toxicity in the human body and the environment, and high mechanical performance^1^,^2^,^3^,^4^,^5^,^6^,^7^,^8^,^9^,^10^. Due to a strong interaction between the optically active polymers with opposite configurations, homo-stereocomplex is formed upon blending L-PLA with its enantiomer poly(d-lactic acid) (D-PLA) or in stereo block poly(lactic acid)s (PLAs)^11^,^12^,^13^,^14^,^15^,^16^,^17^. As shown in Fig. 2^18^, in homo-stereocomplex crystallites, L-PLA and D-PLA segments with opposite configurations or helical directions are packed side-by-side. Homo-stereocomplex formation is also reported for enantiomeric substituted PLAs: poly(2-hydroxybutanoic acid) (P2HB)^19^,^20^ and poly(2-hydroxy-3-methylbutanoic acid) (P2H3MB) (Fig. 1)^21^,^22^. Other examples which can form homo-stereocomplex crystallites include enantiomeric polymer pairs of polyester: poly(*β*-propiolactone)^23^,^24^, polyamides: poly(γ-alkyl glutamate)^25^, poly(hexamethylene di-*O*-methyl tartaramide)^26^,^27^,^28^, polycarbonate: poly(propylene carbonate)^29^, polyether: poly(*tert*-butylene oxide)^30^, polythioether: poly(*tert*-butylene sulfide)^31^, polyketones: poly(propylene-*alt*-carbon monoxide) and poly(1-butene-*alt*-carbon monoxide)^32^, and poly(ester-ether): poly(propylene succinate)^33^. In the case of PLA, the mechanical performance, thermal/hydrolytic degradation-resistance of stereocomplexed materials are higher than those of constituent polymers, L-PLA and D-PLA^11^,^12^,^13^,^14^,^15^,^16^,^17^. A variety of stereo block^34^,^35^,^36^,^37^,^38^,^39^,^40^,^41^,^42^,^43^,^44^,^45^,^46^,^47^,^48^,^49^,^50^,^51^, star-shaped^52^,^53^,^54^,^55^,^56^,^57^,^58^,^59^,^60^,^61^,^62^,^63^,^64^,^65^, star-shaped stereo block PLAs^66^,^67^,^68^,^69^,^70^ were synthesized, and the effects of stereo block and star-shaped or branching architectures on crystallization were extensively investigated and found to have crucial effects on stereocomplex (SC) and homo-crystallization behavior.

On the other hand, SC between the polymers with different chemical structures and opposite configurations, i.e., hetero-stereocomplexes (HTSCs) are formed between two oppositely configured polyesters: PLA and P2HB^71^,^72^, P2HB and P2H3MB^73^,^74^, oppositely configured polyester and polypeptides: d-configured D-PLA and l-configured polypeptides^75^,^76^,^77^,^78^,^79^,^80^,^81^,^82^,^83^,^84^, and oppositely configured polyketones: poly(propylene-*alt*-carbon monoxide) and poly(1-butene-*alt*-carbon monoxide)^32^. Ternary stereocomplex formation takes place in three optically active polyesters: enantiomeric P2HBs and either L-PLA or D-PLA, wherein the polymers with *two different chemical structures* are contained^85^,^86^. Quaternary stereocomplex occurs in four polymers: enantiomeric PLAs and enantiomeric P2HBs, wherein also the polymers with *two different chemical structures* are incorporated^87^. Stereocomplexation occurs in oppositely configured random copolyesters: l- and d-configured poly(2-hydroxybutanoic acid-*co*-lactic acid), which comprise the monomer units with *two different chemical structures*^88^.

As stated above, the stereocomplexation was observed for the blends up to quaternary polymers or monomer units. Although the reported polymer blends which form SC crystallites contain the polymers with *the identical or two different chemical structures*, a stereocomplexationable polymer blend which comprises the polymers with *three or more different chemical structures* has not reported so far. This article reports for the first time an example of a stereocomplexationable polymer blend with *three different chemical structures*. This novel stereocomplexation or co-crystallization strongly suggests that an optically active polymer (l-configured or d-configured polymer) like optically active unsubstituted or substituted PLAs can act as “a configurational or helical molecular glue” for two oppositely configured optically active polymers (two d-configured polymers or two l-configured polymers) which cannot co-crystallize themselves to allow to co-crystallize in one SC crystalline lattice. The combination of l-configured polymer as a configurational or helical molecular glue with at least two d-configured polymers and vice versa will provide a novel way of designing polymeric composites, wherein SC-type co-crystallization will enhance the mechanical properties as reported for L-PLA/D-PLA homo-stereocomplex^11^,^12^,^13^,^14^,^15^,^16^,^17^, and physical properties, and biodegradation rate and behavior can be minutely manipulated.

## Results and Discussion

### Wide-angle X-ray diffractometry

**Figure 3:**
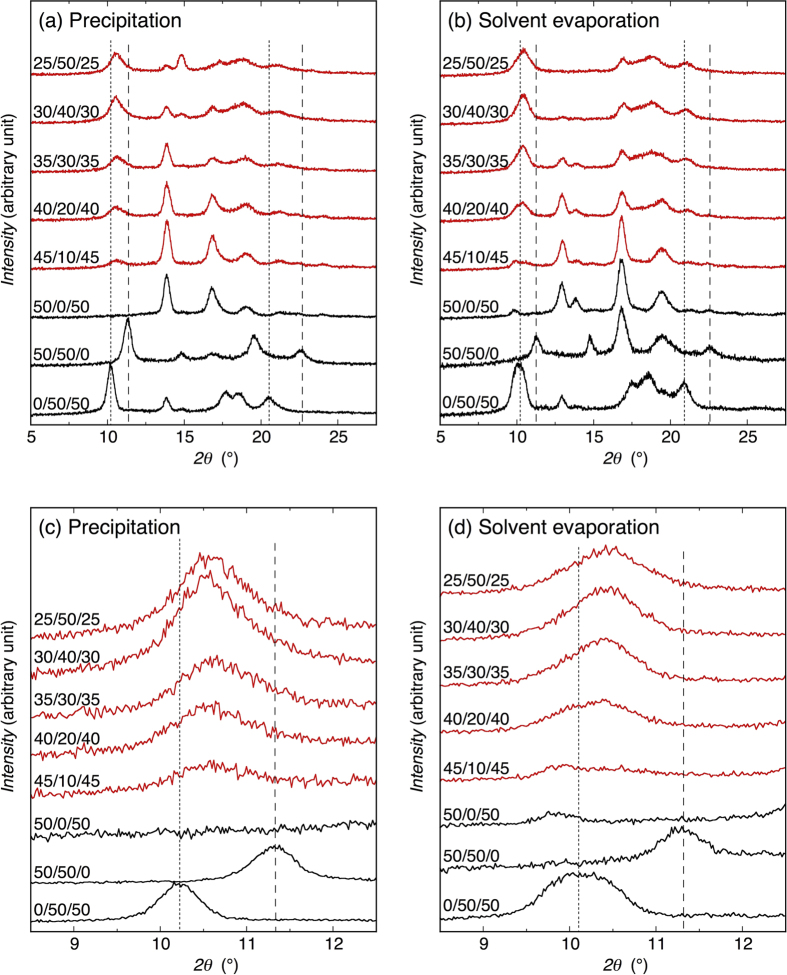
WAXD profiles of blends crystallized by precipitation (**a**,**c**) and solvent evaporation (**b**,**d**). Panels (c) and (d) are magnified figures of panels (a) and (b), respectively, in the *2θ* range of 8.5–12.5°. Shown ratios are those of D-PLA/L-P2HB/D-P2H3MB (mol/mol/mol). Dotted and broken lines indicate the crystalline diffraction angles for L-P2HB/D-P2H3MB and D-PLA/L-P2HB HTSC crystallites, respectively.

For the estimation of crystalline species, interplanar distance (*d*), and crystallinity (*X*_c_) of the blends, wide-angle X-ray diffractometry (WAXD) was performed. Figure 3(a,b) show the WAXD profiles of the blends crystallized by precipitation and solvent evaporation and Fig. 3(c,d) are those magnified in the *2θ* range of 8.5–12.5°. The shown ratios in the figure are those of D-PLA/L-P2HB/D-P2H3MB (mol/mol/mol). For precipitated 0/50/50 blend, i.e., precipitated L-P2HB/D-P2H3MB 50/50 blend, L-P2HB/D-P2H3MB HTSC crystalline peaks were observed at 10.2, 17.7, 18.5, and 20.4° 73,74 and D-P2H3MB and L-P2HB homo-crystalline peaks were seen at 13.8 and 14.9°, respectively^22^. For solvent evaporated 0/50/50 blend, in addition to L-P2HB/D-P2H3MB HTSC crystalline peaks which appeared at the *2θ* values similar to those of precipitated 0/50/50 blend, D-P2H3MB homo-crystalline peaks appeared at 12.9 and 14.0°^22^ and no L-P2HB homo-crystalline peak was observed. Such two different series of *2θ* values were observed for D-P2H3MB homo-crystallites depending on the crystallization method of neat D-P2H3MB samples such as solvent evaporation and melt-crystallization^22^. The precipitated neat D-P2H3MB had the similar diffraction pattern with that reported for melt-crystallized neat D-P2H3MB^22^. For precipitated 50/50/0 blend, i.e., precipitated D-PLA/L-P2HB 50/50 blend, D-PLA/L-P2HB HTSC crystalline peaks were observed at 11.3, 19.5, and 22.5° 71,72 and L-P2HB and D-PLA homo-crystalline peaks were seen at 14.8 and 16.7° 89,90,91, respectively. For solvent evaporated 50/50/0 blend, D-PLA/L-P2HB HTSC crystalline peaks and L-P2HB and D-PLA homo-crystalline peaks appeared at the *2θ* values similar to those of precipitated 50/50/0 blend, although the relative peak heights and areas varied depending on the crystallization method. In summary, in L-P2HB/D-P2H3MB 50/50 blends and D-PLA/L-P2HB 50/50 blends, l-configured L-P2HB can form similar HTSC crystallites with d-configured D-P2H3MB or D-PLA.

For 50/0/50 blends, i.e., D-PLA/D-P2H3MB 50/50 blends comprising only d-configured polymers, it is expected that D-PLA and D-P2H3MB homo-crystallites are separately formed in the blend. As expected, for precipitated 50/0/50 blend, D-P2H3MB homo-crystalline peaks were explicitly observed at 13.8, 21.2, and 24.0° 22 and D-PLA homo-crystalline peaks were seen at 16.8, 19.0, and 22.5° 89,90,91. Other D-P2H3MB homo-crystalline peaks at 17.1, 18.9, and 21.8° should be included in large D-PLA homo-crystalline peaks. In the case of solvent evaporated 50/0/50 blend, although D-PLA homo-crystalline peaks were observed at the similar *2θ* values, D-P2H3MB homo-crystalline peaks were explicitly observed at 9.8, 12.9, 13.8, and 21.3° 22 and another D-P2H3MB homo-crystalline peak at 16.8° should be contained in large D-PLA homo-crystalline peaks^89^,^90^,^91^.These results exhibit that both d-configured polymers, D-PLA and D-P2H3MB, crystallized separately to form their homo-crystallites in 50/0/50 blends, which were composed of only d-configured polymers.

For the ternary D-PLA/L-P2HB/D-P2H3MB blends (red profiles in Fig. 3), which were composed of two d-configured polymers and one l-configured polymer, in addition to the D-PLA and D-P2H3MB homo-crystalline peaks, a new crystalline peak appeared at around 10.5° and its peak intensity increased with increasing L-P2HB content [Fig. 3(c,d)]. This new crystalline peak was located between the main crystalline peaks of D-PLA/L-P2HB HTSC crystallites (broken lines) and L-P2HB/D-P2H3MB HTSC crystallites (dotted lines) in 50/50/0 and 0/50/50 blends, respectively, and was not observed for D-PLA, L-P2HB, and D-P2H3MB homo-crystallites. These results strongly suggest that the peak at around 10.5° for the ternary polymer blends can be ascribed to SC crystallites. As seen in magnified WAXD profiles [Fig. 3(c,d)], the superposition of main peaks of D-PLA/L-P2HB HTSC crystallites and L-P2HB/D-P2H3MB HTSC crystallites in 50/50/0 and 0/50/50 blends, respectively, cannot form the crystalline peaks observed in the ternary polymer blends in the *2θ* range of 8.5–12.5°.

Also, the crystalline peak observed at around 21.1° became higher with increasing L-P2HB content, i.e., decreasing D-P2H3MB content in ternary polymer blends. At low L-P2HB contents or high D-P2H3MB contents, the crystalline peak observed at around 21.1° can be ascribed to D-P2H3MB homo-crystallites, whereas for a high L-P2HB content or a low D-P2H3MB content, this peak cannot be attributed to D-P2H3MB homo-crystallites or other homo-crystallites but can be ascribed to SC crystallites. Normally, other SC crystalline peaks can be observed in the *2θ* range of 10.5–21.1°. However, there were many intense crystalline peaks in this *2θ* range, other SC crystalline peaks should have been contained in or overlapped with other intense crystalline peaks and, therefore, other SC crystalline peaks could not be observed in the *2θ* range of 10.5–21.1°, independently. With an increase in L-P2HB content, the D-P2H3MB homo-crystalline peaks at 13.8 and 24.0° and D-PLA homo-crystalline peak at 16.8° became smaller in the precipitated ternary blends, and the D-P2H3MB homo-crystalline peaks at 9.8, 12.9 and 13.8° and D-PLA homo-crystalline peak at 16.8° got smaller in the solvent evaporated ternary blends. These results support the SC formation in the ternary blends. The crystalline peaks observed at 14.8° for precipitated 30/40/30 and 25/50/25 blends can be ascribed to L-P2HB homo-crystallites.

**Figure 4:**
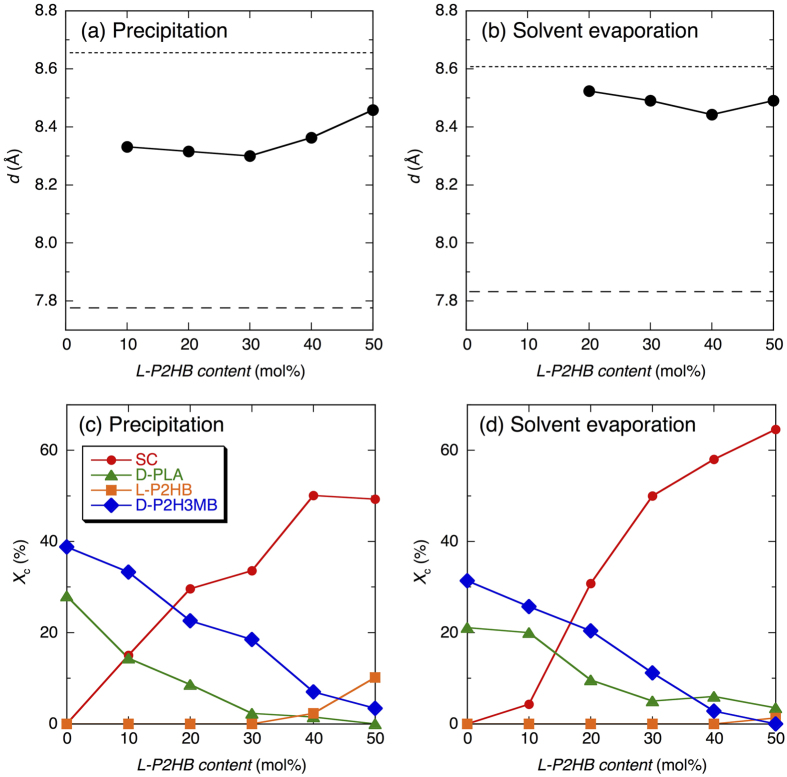
Interplanar distance (*d*) of SC crystallites in ternary polymer blends crystallized by precipitation (**a**) and solvent evaporation (**b**) for *2θ* range of 8.5–12.5°, crystallinity (*X*_c_) of 50/0/50 and ternary polymer blends crystallized by precipitation (**c**) and solvent evaporation (**d**). Dotted and broken lines in panels (a) and (b) indicate the *d* values for L-P2HB/D-P2H3MB HTSC crystallites in 0/50/50 blends and D-PLA/L-P2HB HTSC crystallites in 50/50/0 blends, respectively.

The *d* values of SC crystallites in ternary polymer blends for *2θ* range of 8.5–12.5° were estimated from the WAXD profiles in Fig. 3 and are plotted in Fig. 4(a) and (b) as a function of L-P2HB content. Due to strong overlapping of SC crystalline peak and D-P2H3MB homo-crystalline peak, *d* could not be estimated for solvent evaporated blends at L-P2HB content of 10 mol%. As seen in Fig. 4, *d* values of SC crystallites in ternary polymer blends were between those of L-P2HB/D-P2H3MB HTSC crystallites (dotted lines) in 0/50/50 blends and D-PLA/L-P2HB HTSC crystallites (broken lines) in 50/50/0 blends. For the *2θ* range of 8.5–12.5°, the *d* values of precipitated and solvent evaporated blends at around 8.3 and 8.5 Å, respectively, were intermediate between the *d* values for L-P2HB/D-P2H3MB HTSC crystallites in 0/50/50 blends and D-PLA/L-P2HB HTSC crystallites in 50/50/0 blends and were correspondingly slightly and very close to the *d* value of L-P2HB/D-P2H3MB HTSC crystallites.

The *X*_c_ values of blends were estimated from the WAXD profiles in Fig. 3. The thus obtained *X*_c_ values are summarized in Table S1 in Supporting Information and those of 50/0/50 and ternary blends are plotted in Fig. 4(c) and (d) as a function of L-P2HB content. As seen in Fig. 4(c) and (d), in both precipitated and solvent evaporated blends, *X*_c_ values of SC crystallites increased but *X*_c_ values of homo-crystallites of d-configured D-PLA and D-P2H3MB decreased with increasing L-P2HB content.

### Differential scanning calorimetry

**Figure 5:**
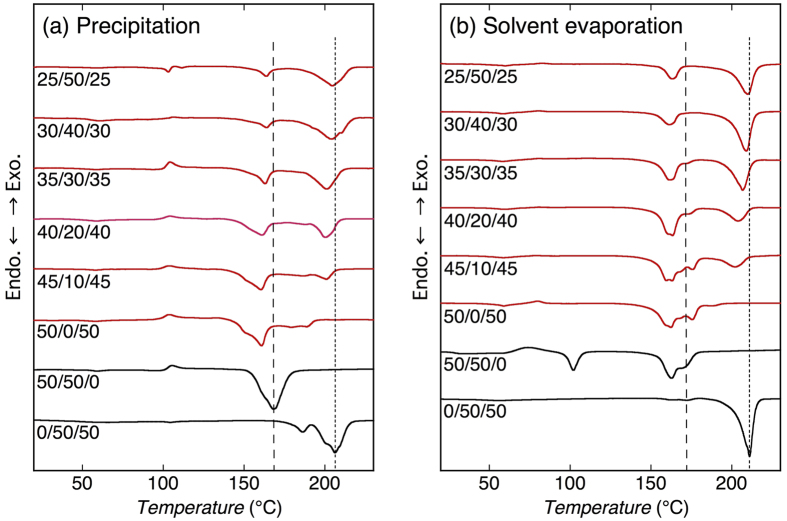
DSC thermograms of blends crystallized by precipitation (**a**) and solvent evaporation (**b**). Shown ratios are those of D-PLA/L-P2HB/D-P2H3MB (mol/mol/mol). Dotted and broken lines in panels (a) and (b) indicate the *T*_m_ values for L-P2HB/D-P2H3MB HTSC crystallites in 0/50/50 blends and D-PLA/L-P2HB HTSC crystallites in 50/50/0 blends, respectively.

For the estimation of thermal properties of the blends, differential scanning calorimetry (DSC) was carried out (Fig. 5). The thermal properties estimated from the DSC thermograms in Fig. 5 are summarized in Table S2 in Supporting Information. For precipitated 0/50/50 blend, three melting peaks of L-P2HB and D-P2H3MB homo-crystallites and L-P2HB/D-P2H3MB HTSC crystallites were observed at 104, 186, and 206 °C, respectively. Although due to the overlapping of melting peaks, separate estimation of melting enthalpy (*ΔH*_m_) values of respective crystalline species could not be performed, peak area was the largest for L-P2HB/D-P2H3MB HTSC crystallites, in agreement with the WAXD result. Solvent evaporated 0/50/50 blend showed similar DSC thermograms, although melting peak of L-P2HB homo-crystallites was not observed. For precipitated 50/50/0 blend, melting peaks were observed at 95 and 168 °C. The former is attributable to melting peak of L-P2HB homo-crystallites and the latter can be assigned to overlapped melting of D-PLA homo-crystallites and D-PLA/L-P2HB HTSC crystallites. However, on the basis of a large *X*_c_ value of D-PLA/L-P2HB HTSC crystallites, most of the latter peak should have been mainly composed of melting of HTSC crystallites. For solvent evaporated 50/50/0 blend, the melting peaks of L-P2HB and D-PLA homo-crystallites and D-PLA/L-P2HB HTSC crystallites appeared at 102, 163, and 168 °C, respectively. The melting temperature (*T*_m_) values of L-P2HB/D-P2H3MB HTSC crystallites and D-PLA/L-P2HB HTSC values are consistent with the reported values^71^,^72^,^73^,^74^.

For precipitated 50/0/50 blend comprising only d-configured D-PLA and D-P2H3MB, melting peaks of D-PLA and D-P2H3MB homo-crystalline peaks were observed at 161 °C and 179 and 189 °C, respectively, whereas for solvent evaporated 50/0/50 blend, melting peaks of D-PLA and D-P2H3MB homo-crystalline peaks were seen at 162 and 176 °C, respectively. For ternary blends, in addition to the melting peaks of D-PLA and D-P2H3MB homo-crystallites, a new melting peak appeared at around 200 °C and its intensity or area increased with increasing L-P2HB content, indicating this peak is attributable to the melting of SC crystallites.

## Discussion

The SC-type crystalline peaks in WAXD profiles (Fig. 3) and crystallinity of SC-type crystallites [Fig. 4(c) and (d)] of the ternary polymer blends increased with an increase in L-P2HB content. Furthermore, for the ternary polymer blends, the new higher melting peak appeared in DSC thermograms and its intensity or area increased with increasing L-P2HB content (Fig. 5). These results indicate the formation of SC crystallites in ternary polymer blends. In the ternary polymer blends, two types of HTSC crystallites, i.e., D-PLA/L-P2HB HTSC crystallites and L-P2HB/D-P2H3MB HTSC crystallites can be formed. As evident from Fig. 3(c) and (d), the superposition of WAXD profiles of D-PLA/L-P2HB and L-P2HB/D-P2H3MB HTSC crystallites in 50/50/0 and 0/50/50 blends, respectively, cannot form the SC crystalline peaks observed in the ternary polymer blends. Moreover, the *d* values in the *2θ* range of 8.5–12.5° were between those of D-PLA/L-P2HB HTSC crystallites and L-P2HB/D-P2H3MB HTSC crystallites [Fig. 4(a) and (b)]. These results deny the separate formation of two types of L-P2HB/D-P2H3MB HTSC crystallites and D-PLA/L-P2HB HTSC crystallites and indicate the formation of ternary stereocomplex crystallites which contain two different d-configured D-PLA and D-P2H3MB and l-configured L-P2HB. In other words, incorporated L-P2HB allowed non-cocrystallizable d-configured D-PLA and D-P2H3MB to co-crystallize in one SC crystallites by the attractive interaction of l-configured L-P2HB with d-configured D-PLA and D-P2H3MB. Table 1 tabulates the reported SCs of unsubstituted and substituted PLAs, together with the types of polymer chain and chemical structure. Previously, we reported HTSC, ternary stereocomplex, and quaternary stereocomplex formation of two, three, and four homopolymers, respectively, but these SCs comprise the optically active homopolymers with up to only *two different chemical structures*. However, as evident from Table 1, this article reports for the first time SC formation from optically active homopolymers with *three different chemical structures*.

For the *2θ* range of 8.5–12.5°, the *d* values of ternary stereocomplex crystallites in the precipitated ternary blends were slightly closer to the *d* value of L-P2HB/D-P2H3MB HTSC crystallites than that of D-PLA/L-P2HB HTSC crystallites, whereas the *d* values of ternary stereocomplex of the solvent evaporated ternary blends was much closer to the *d* value of L-P2HB/D-P2H3MB HTSC crystallites than that for D-PLA/L-P2HB HTSC crystallites [Fig. 4(a) and (b)]. These results are indicative of the fact that ternary stereocomplex crystallites contain a higher amount of larger sized d-configured D-P2H3MB and a lower amount of small sized d-configured D-PLA and the attractive force of l-configured L-P2HB during precipitation and solvent evaporation acted correspondingly slightly and much stronger for d-configured D-P2H3MB than for d-configured D-PLA. These results are consistent with the fact that HTSC formation occurs readily between L-P2HB and D-P2H3MB compared to that between D-PLA and L-P2HB^71^,^72^,^73^,^74^.

**Figure 6:**
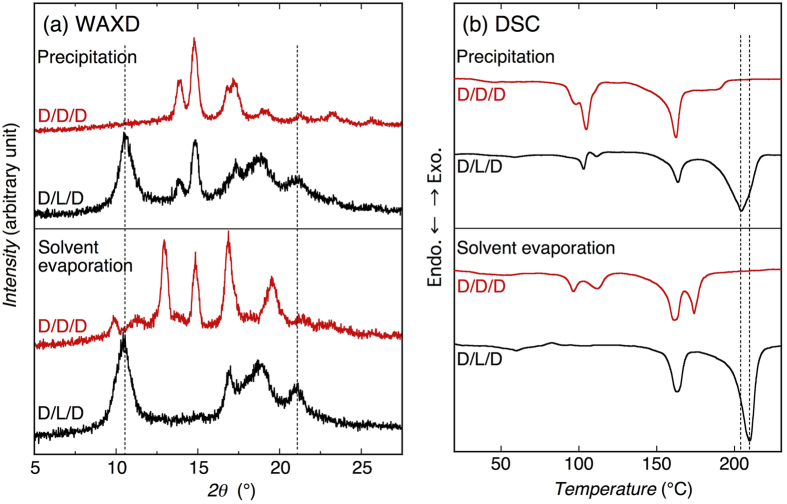
WAXD profiles (**a**) and DSC thermograms (**b**) of D-PLA/D-P2HB/D-P2H3MB (25/50/25) (D/D/D) and D-PLA/L-P2HB/D-P2H3MB (25/50/25) (D/L/D) blends crystallized by precipitation and solvent evaporation. Dotted lines in panel (a) are representative diffraction angles of ternary stereocomplex crystallites and those in panel (b) are *T*_m_ values of ternary stereocomplex crystallite

Here, we must consider the probability that not l-configured L-P2HB but d-configured D-P2HB having the same configuration with that of d-configured D-PLA and D-P2H3MB may act a glue and form the co-crystallites in D-PLA/D-P2HB/D-P2H3MB ternary polymer blends. To exclude the probability, all d-configured ternary D-PLA/D-P2HB/D-P2H3MB (25/50/25) blends (abbreviated as D/D/D blends) were prepared and their crystallization behavior was investigated by WAXD and DSC. The obtained WAXD profiles and DSC thermograms are shown in Fig. 6, together with those of ternary D-PLA/L-P2HB/D-P2H3MB (25/50/25) blends (abbreviated as D/L/D blends) for reference. It is evident that the crystalline diffraction peaks in WAXD profiles and melting peak in DSC thermograms, which are attributable to a new type of co-crystallites, were not observed for all d-configured D/D/D blends. This result confirms that the only l-configured L-P2HB can attract d-configured D-PLA and D-P2H3MB and facilitate co-crystallization of d-configured D-PLA and D-P2H3MB to form ternary stereocomplex crystallites.

**Figure 7:**
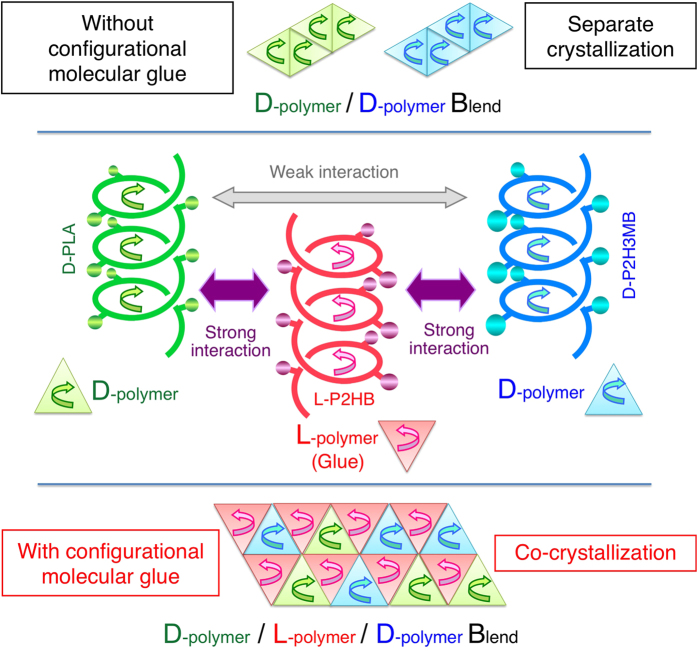
Schematic representation of separate crystallization of D-configured D-PLA and D-P2H3MB and co-crystallization of D-PLA and D-P2H3MB by helical or configurational molecular glue of L-configured L-P2HB.

This article reports a very interesting result that l-configured L-P2HB attracts d-configured D-PLA and D-P2H3MB, which will not co-crystallize in a crystalline lattice without l-configured L-P2HB, to co-crystallize into ternary stereocomplex crystallites. Since, l-configured L-PLA have the helical structure with its direction opposite with that of d-configured D-PLA in homo-stereocomplex crystallites^18^, l-configured substituted PLAs, L-P2HB and P(L-2H3MB), are expected to have the helical structures with their directions opposite to d-configured substituted PLAs, D-P2HB and D-P2H3MB. Therefore, the results obtained in the present article strongly suggests that an optically active polymer (l-configured or d-configured polymer) like optically active unsubstituted or substituted PLAs can act as a configurational or helical molecular glue for two oppositely configured optically active polymers (two d-configured polymers or two l-configured polymers) which cannot co-crystallize themselves to allow to co-crystallize in one ternary stereocomplex crystalline lattice, as schematically illustrated in Fig. 7. The structure of ternary stereocomplex here can be regarded as cardboard boxes (composed of three L-P2HB chains) which can house slightly different sized bottles (one D-PLA or D-P2H3MB chain) and shield two types of D-polymers. However, the present system differs from the so-called “unbalanced packing of chiral low molecular weight molecules” 93,94 which associates one L-isomer with two D-isomers (all of the same species) and has three entities with a fixed ratio of one to two in the unit-cell. The increased degree of freedom in polymer combination in the present study is expected to assist to pave the way for designing polymeric composites having a wide variety of physical properties, biodegradation rate and behavior in the case of biodegradable polymers.

## Method

### Materials

D-PLA, L-P2HB, D-P2HB, and D-P2H3MB were synthesized by polycondensation of d-lactic acid, l-2-hydroxybutanoic acid [(*S*)-2-hydroxybutyric acid] (≥97.0%, Sigma-Aldrich Co., Tokyo, Japan), d-2-hydroxybutanoic acid [(*R*)-2-hydroxybutyric acid] (≥98.0%, Sigma-Aldrich Co.), and d-2-hydroxy-3-methylbutanoic acid [(*R*)-2-hydroxy-3-methylbutyric acid or d-*α*-hydroxyisovaleric acid] (≥98.0%, Sigma-Aldrich Co.), using 5 wt% *p*-toluenesulfonic acid (monohydrate, JIS special grade, Nacalai Tesque inc., Kyoto, Japan) as the catalyst, as reported previously^19^,^22^,^92^. d-lactic acid was prepared by hydrolytic degradation of d-lactide (assay 99.5%, Purac Biochem, Gorinchem, The Netherlands) with distilled water (Special grade for HPLC, Nacalai Tesque inc.) [d-lactide/water (mol/mol) = 1/2] at 98 °C for 30 min. The polycondensation reaction of monomers was performed at 130 °C under atmospheric pressure for 5 h for the synthesis of all polymers and then under reduced pressure of 1.8 kPa for 24 h for the synthesis of D-PLA, of 2.0 kPa for 24 h for the synthesis of L-P2HB, of 1.6 kPa for 6 h for the synthesis of D-P2HB, and of 1.4 kPa for 12 h for the synthesis of L-P2H3MB and D-P2H3MB. The synthesized polymers were purified by reprecipitation using chloroform and methanol (both JIS special grade, Nacali Tesque Inc.) as the solvent and nonsolvent, respectively. The purified polymers were dried under reduced pressure at least 6 days.

Ternary or binary polymer blends were prepared by the procedure stated in the previous papers^11^,^19^,^71^,^85^,^87^. Briefly, each solution of the three or two polymers was prepared separately to have a polymer concentration of 1.0 g dL^−1^ and then admixed with each other under vigorous stirring. Dichloromethane (JIS special grade, Nacali Tesque Inc.) was used as the solvent. The mixed solution was cast onto a petri-dish, followed by solvent evaporation at 25 °C for approximately one day. The obtained blends were further dried under reduced pressure at least 6 days. The precipitated blends were obtained by dissolving solution-cast blends using dichloromethane as the solvent to have a polymer concentration of 10 g dL^−1^ and reprecipitation with stirred methanol as the nonsolvent. The volume ratio of blend solution and methanol 0.5/30 (mL/mL). The precipitated blends were rinsed with fresh methanol twice and dried under reduced pressure for at least 6 days.

### Physical measurements and observation

The weight- and number-average molecular weights (*M*_w_ and *M*_n_, respectively) of the polymers were evaluated in chloroform at 40 °C using a Tosoh (Tokyo, Japan) gel permeation chromatography system with two TSK gel columns (GMH_XL_) and polystyrene standards. Therefore, the *M*_w_ and *M*_n_ values are given relative to polystyrene. The specific optical rotation ([*α*]^25^_589_) of the polymers was measured in chloroform at a concentration of 1 g dL^−1^ and 25 °C using a JASCO (Tokyo, Japan) P-2100 polarimeter at a wave length of 589 nm. The glass transition, cold crystallization, and melting temperatures (*T*_g_, *T*_cc_, and *T*_m_, respectively) and the enthalpies of cold crystallization and melting (*ΔH*_cc_ and *ΔH*_m_, respectively) were determined with a Shimadzu (Kyoto, Japan) DSC-50 differential scanning calorimeter under a nitrogen gas flow at a rate of 50 mL^ ^min^−1^. The samples (ca. 3 mg) were heated from 0 to 250 °C at a rate of 10 °C^ ^min^−1^. Wide-angle X-ray diffractometry was carried out at 25 °C using a RINT-2500 (Rigaku Co., Tokyo, Japan) equipped with a Cu-K*α* source [wave length (λ) = 1.5418 Å]. Molecular characteristics of the polymers used in the present study are shown in Table 2.

## Additional Information

**How to cite this article:** Tsuji, H. *et al*. Configurational Molecular Glue: One Optically Active Polymer Attracts Two Oppositely Configured Optically Active Polymers. *Sci. Rep.* **7**, 45170; doi: 10.1038/srep45170 (2017).

**Publisher's note:** Springer Nature remains neutral with regard to jurisdictional claims in published maps and institutional affiliations.

## Supplementary Material

Supplementary Information

## Figures and Tables

**Figure 1:**
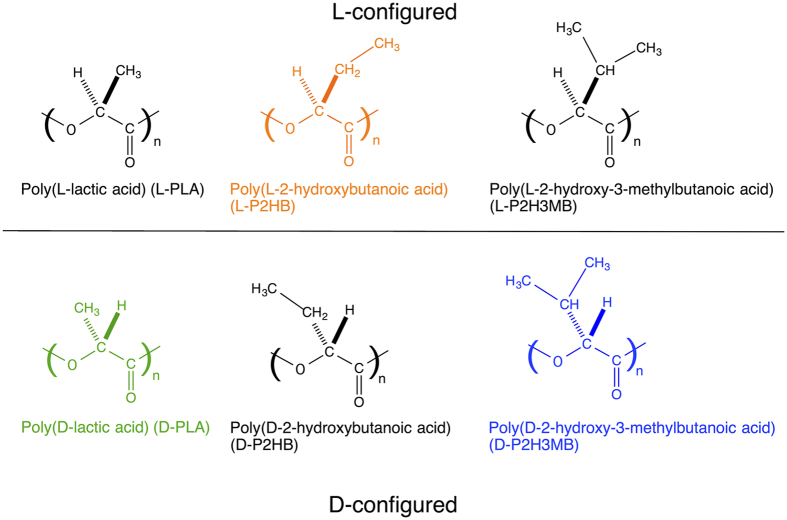
Molecular structures of unsubsitued and substituted PLAs.

**Figure 2:**
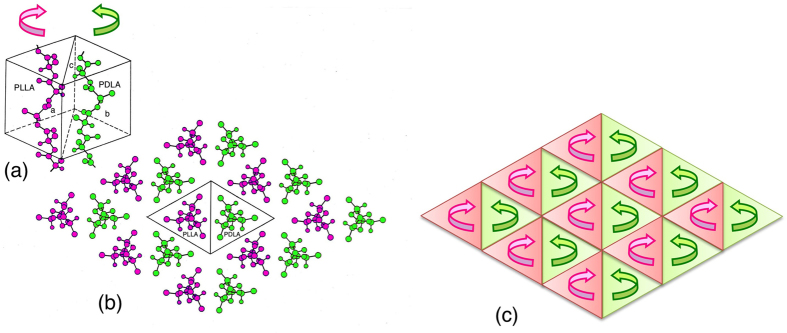
Structural model of PLA SC (**a**), molecular arrangement (**b**) and helical direction of PLA chains (b) projected on the plane normal to the chain axis. The arrows indicate the relative directions of PLA helices. Panels (a) and (b) are Reprinted from ref. 18, T. Okihara, *et al*., *J. Macomol Sci. Part B: Phys.,* vol. B30, 735-736, Crystal structure of stereocomplex of poly(L-lactide) and poly(D-lactide), pp. 119–140, Copyright (1991), with permission from Taylor & Francis. In panels (a) and (b), L-PLA and D-PLA are abbreviated as PLLA and PDLA, respectively. In panel (a), the arrows are added to original figure and in panel (b) a line between L-PLA and D-PLA is added.

**Table 1:** Features of reported SCs of unsubstituted and substituted PLAs.

Crystalline species	Blend	Type of polymer chain	Type of chemical structure	Reference
Homo-stereocomplex	L-PLA/D-PLA	2	1	11
	L-P2HB/D-P2HB	2	1	19
	L-P2H3MB / D-P2H3MB	2	1	21
HTSC	L-P2HB/D-PLA	2	2	71
	L-P2HB/D-P2H3MB	2	2	73
Ternary stereocomplex	L-PLA or D-PLA/L-P2HB/D-P2HB	3	2	85
	D-PLA/L-P2HB/D-P2H3MB	3	3	Present study
Quaternary stereocomplex	L-P2HB/D-P2HB/L-P2H3MB/D-P2H3MB	4	2	87

**Table 2:** Molecular characteristics of polymers used in the present study.

Polymer	*M*_w_^a)^ (g mol^−1^)	*M*_w_/*M*_n_^a)^	[*α*]^25^_589_^b)^ (deg dm^−1^ g^−1^ cm^3^)
D-PLA	1.90 × 10^4^	1.44	158.1
L-P2HB	2.29 × 10^4^	1.60	−116.1
D-P2HB	1.12 × 10^4^	1.95	116.2
D-P2H3MB	4.11 × 10^3^	1.92	88.0

^a^*M*_w_ and *M*_n_ are weight- and number-average molecular weights, respectively, estimated by GPC.

^b^Measured in chloroform
