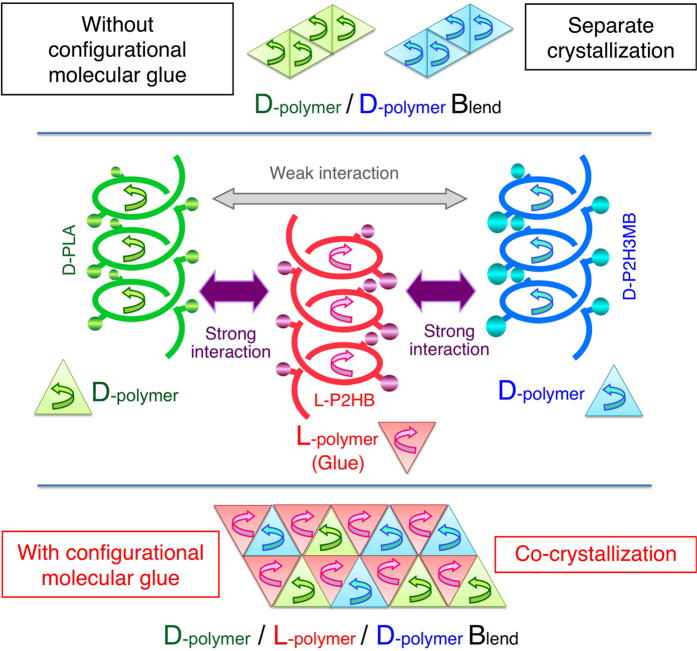# Corrigendum: Configurational Molecular Glue: One Optically Active Polymer Attracts Two Oppositely Configured Optically Active Polymers

**DOI:** 10.1038/srep46989

**Published:** 2018-05-22

**Authors:** Hideto Tsuji, Soma Noda, Takayuki Kimura, Tadashi Sobue, Yuki Arakawa

Scientific Reports 7: Article number: 4517010.1038/srep45170; published online: 03 24 2017; updated: 05 22 2018

This Article contains an error in Figure 7, where the arrows indicating the helical directions are inconsistent with Figure 2. The correct Figure 7 appears below as Figure 1.

## Figures and Tables

**Figure 1:**