# Supplementary material for: Configurational Molecular Glue: One Optically Active Polymer Attracts Two Oppositely Configured Optically Active Polymers
Source: Sci Rep. 2017 Mar 24;7:45170. doi: 10.1038/srep45170 (PMC5364539; doi:10.1038/srep45170)
Supplement: Supplementary Information [file srep45170-s1.pdf]

Supplementary Information for:

Configurational Molecular Glue: One Optically Active Polymer Attracts Two Oppositely Configured Optically Active Polymers

Hideto Tsuji, Soma Noda, Takayuki Kimura, Tadashi Sobue, and Yuki Arakawa*

Department of Environmental and Life Sciences,

Graduate School of Engineering,

Toyohashi University of Technology,

Tempaku-cho, Toyohashi, Aichi 441-8580, Japan

E-mail: tsuji@ens.tut.ac.jp

Contents:

S1. Crystallinity values of blends (Table S1)

S2. Thermal properties of blends (Table S2)

S1. Crystallinity values of blends

Table S1. Crystallinity values of blends.

Crystallization method	D-PLA/L-P2HB/D-P2H3MB (mol/mol/mol)	Type of SC	X_c(SC) (%)	X_c(D-PLA) (%)	X_c(L-P2HB) (%)	X_c(D-P2H3MB) (%)	X_c(tot) [a] (%)
precipitation	0/50/50	HTSC	61.1	-	1.6	8.1	70.8
	50/50/0	HTSC	52.0	3.8	2.2	-	58.0
	50/0/50		-	28.0	-	38.8	66.8
	45/10/45	TSC [b]	15.0	14.4	-	33.3	62.7
	40/20/40	TSC [b]	29.6	8.6	-	22.6	60.8
	35/30/35	TSC [b]	33.6	2.3	-	18.5	54.4
	30/40/30	TSC [b]	50.1	1.5	2.3	7.0	60.9
	25/50/25	TSC [b]	49.3	-	10.2	3.4	62.9
Solvent evaporation	0/50/50	HTSC	70.5	-	-	7.3	77.8
	50/50/0	HTSC	23.6	19.2	5.3	-	48.1
	50/0/50		-	21.1	-	31.4	52.5
	45/10/45	TSC [b]	4.3	20.0	-	25.7	50.0
	40/20/40	TSC [b]	30.8	9.6	-	20.4	60.8
	35/30/35	TSC [b]	50.0	5.0	-	11.2	66.2
	30/40/30	TSC [b]	58.0	6.0	-	2.8	66.8
	25/50/25	TSC [b]	64.6	3.5	1.3	-	69.4

[a] X_c(tot) = X_c(SC) + X_c(D-PLA) + X_c(L-P2HB) + X_c(D-P2H3MB).
[b] Ternary stereocomplex.

S2. Thermal properties of blends

Table S2. Thermal properties of blends.

Crystallization method	D-PLA/L-P2HB/D-P2H3MB (mol/mol/mol)	T_g [a] (°C)	T_{cc} [a] (°C)	T_m [a] (°C)	ΔH_{cc} [b] (J g^{-1})	ΔH_m [b] (J g^{-1})	ΔH(tot) [b] (J g^{-1})
precipitation	0/50/50	32.8	-	104.2, 186.2, 206.1	-	41.9	41.9
	50/50/0	54.7	105.5	95.1, 168.2 [c]	-1.8	47.4	45.6
	50/0/50	27.3, 48.3	103.8, 133.6	160.7, 179.0, 188.6	-7.7	39.0	31.3
	45/10/45	40.1, 51.6	103.9	160.3, 186.8, 200.7	-3.4	41.3	37.9
	40/20/40	44.8	103.9	160.9, 187.9, 200.5	-3.6	42.7	39.1
	35/30/35	42.7	104.1	92.9, 163.0, 200.9	-3.7	41.9	38.2
	30/40/30	50.7	106.5	163.7, 204.1	-1.3	43.8	42.5
	25/50/25	33.1, 51.1	-	103.0, 111.5, 163.5, 204.4	-	38.2	38.2
Solvent evaporation	0/50/50	42.6		162.8, 171.6, 210.8	-	-	45.5
	50/50/0	25.3	73.8	101.9, 162.7, 168.4	-7.8	43.1	35.3
	50/0/50	52.8	79.6	162.1, 175.6	-1.6	43.5	41.9
	45/10/45	49.0	79.0	159.5, 163.0, 175.5, 201.9	-0.2	51.8	51.6
	40/20/40	49.5	80.8	160.7, 163.3, 203.9	-0.1	46.9	46.8
	35/30/35	46.4	81.8	161.4, 206.4	-0.2	49.0	48.8
	30/40/30	50.0	80.7	161.0, 208.8	-0.5	50.5	50.0
	25/50/25	42.5	82.6	163.0, 209.6	-0.9	47.1	46.2

[a] T_g, T_{cc}, and T_m are glass transition, cold crystallization, and melting temperatures, respectively.
[b] ΔH_{cc} and ΔH_m are cold crystallization and melting enthalpies, respectively. ΔH(tot) = ΔH_{cc} + ΔH_m.
[c] Melting peak of D-PLA homo-crystallites was included in that of D-PLA/L-P2HB HTSC crystallites.
